# The Role of Waste Management in Control of Rabies: A Neglected Issue

**DOI:** 10.3390/v13020225

**Published:** 2021-02-01

**Authors:** Nicolette Wright, Deepak Subedi, Saurav Pantha, Krishna Prasad Acharya, Louis Hendrik Nel

**Affiliations:** 1 Department of Biochemistry, Genetics and Microbiology, Faculty of Natural and Agricultural Sciences, University of Pretoria, Pretoria 0083, South Africa; louis.nel@up.ac.za; 2 Paklihawa Campus, Institute of Agriculture and Animal Science, Tribhuvan University, Paklihawa, Rupandehi 32900, Nepal; subedideepu26@gmail.com (D.S.); sauravvet@gmail.com (S.P.); 3 Animal Quarantine Office (AQO), Department of Livestock Services, Kathmandu 44600, Nepal; kriaasedu@gmail.com; 4 Global Alliance for Rabies Control SA NPC, Erasmus Forum A434, South Erasmus Rand, Pretoria 0181, South Africa

**Keywords:** municipal waste, free-roaming dogs, rabies control

## Abstract

Despite being vaccine preventable, the global burden of dog rabies remains significant, and historically it is the rural and marginalized communities in developing countries of Africa and Asia that are most threatened by the disease. In recent years, the developing world has been experiencing unprecedented increases in urbanization, with a correspondingly massive increase in municipal solid waste generation, among other things. Inefficient and inadequate waste collection and management, due to lack of resources and planning, led to significant increases in the volumes of waste on the streets and in open dumps, where it serves as food sources for free-roaming dogs. In this commentary, we discuss examples of poor waste management and the likely impact on rabies control efforts through the sustenance of free-roaming dogs in some dog rabies-endemic countries. We aim to stress the importance of implementing strategies that effectively address this particular issue as an important component of humane dog population management, as it relates to aspirations for the control and elimination of dog rabies per se.

## 1. Introduction

Warm-blooded vertebrates, including humans, are susceptible to rabies—a vaccine-preventable and neglected tropical zoonotic disease that is characterized by acute, nearly 100% fatal neurological complications [1,2]. The RNA viruses of the *Lyssavirus* genus, family *Rhabdoviridae,* are all known or suspected to induce rabies, although the main etiological agent for rabies worldwide remains rabies virus (RABV) [3,4]. In spite of being one of the oldest known zoonotic infections, it is still a neglected disease with the greatest burden in rural marginalized communities of developing countries [4,5,6,7,8]. Despite successful control and elimination of dog-mediated rabies in some parts of the world, this disease is still endemic in at least 122 countries [9]. The primary measure adopted to control dog-mediated rabies is mass dog vaccination. It is necessary to maintain the 70% vaccination coverage to provide herd immunity in a dog population in order to break the chain of transmission [10]. However, achieving such vaccination coverage is most often thwarted by rapid turnover in the dog populations resulting in vaccinated populations being replaced by non-vaccinated dogs [11,12,13]. While some overall positive reports on the effect of catch–neuter–vaccinate–release (CNVR) programs, such as increasing the general health of street dog populations as well as assisting in population control, have been published, other studies have continued to report short life-expectancy coupled with rapid population turnover rates in free-roaming dog populations, despite population management interventions [14]. For the purpose of this discussion, free-roaming dogs include owned dogs that are allowed to roam, community owned dogs that are free-roaming, and true stray dogs.

The Food and Agriculture Organization of the United Nations (FAO), the World Organization for Animal Health (OIE), the World Health Organization (WHO), and the Global Alliance for Rabies Control (GARC) in 2015 collectively set the common goal to end dog-mediated rabies deaths by 2030 through mass dog vaccination and rabies awareness programs [15]. A well-coordinated and collaborative approach from all concerned stakeholders is necessary to attain the pre-determined goal. In dog rabies endemic areas, an effective strategic vaccination that includes coverage of at least 70% of the dog population is considered to be crucial [16]. Out of the estimated global population of domestic dogs (687 million), 78% (536 million) are living in 122 dog rabies endemic countries [9,14]. Rapidly growing countries like India and Bangladesh hold higher risks because of increasing urbanization, urban slums, and populations of unvaccinated street dogs living in close proximity to poorly managed garbage disposal sites. Asia and Africa are home to 51.55% and 24.28% of the global dog population and account for around 59.6% and 36.4% of the global deaths due to rabies, respectively [4,16]. India, a home to 32.68% of the Asian dog population, is responsible for about 59.9% and 35% of rabies deaths in Asia and the world, respectively [4,16]. Efforts to attain the desired 70% vaccination coverage in Africa and Asia are often hampered by a lack of political support for rabies elimination, which is then typically reflected in the allocation of limited resources to other prioritized areas. The most developed African and Asian countries achieve vaccination rates of 30–50% of pet animals, while the corresponding vaccination estimates may be much lower in stray dog populations [4,17]. Catch–neuter–vaccinate–release (CNVR) programs, as a part of rabies control programs, have been successfully implemented in Asian rabies endemic countries like Bhutan [14], Sri-Lanka [18], and India [19]. Rabies control programs require a One Health approach, where multiple sectors (i.e., human as well as animal health sectors) collaborate in order to facilitate effective control efforts (where control in the animal host such as dogs facilitates control of the disease in humans). However, progress in the control of dog rabies has mostly been disappointing where strategies were not entirely synchronous as far as the need for a One Health approach is concerned [20].

Global dog population numbers are on the increase, with European countries experiencing between 6% and 7.7% increase in pet dog population size from 2016 to 2019 [21]. Similarly, the dog population in the United States of America (USA) increased by 15.29% from 2015 to 2017 [22], whereas Indian cities have reported a dramatic 65% increase in dog population numbers [23]. Given current trends, a 149% increase in the number of pet dogs in India was predicted from 2014 to 2023 [24]. Where large numbers of free-roaming dogs are present with high dog population turnover rates, it becomes increasingly difficult to reach and maintain the 70% vaccination coverage required for herd immunity [25]. In 2015, it was estimated that 130 million dog rabies vaccines were administered worldwide, 20 million vaccine doses short of a predicted market of 150 million doses. The shortage of available vaccines is expected to increase to 246 million doses if current production capacity is maintained—ultimately leading to fewer dogs being vaccinated overall (based on a goal of 375 million dogs vaccinated against rabies by 2030) [9].

## 2. Urbanization and Solid Waste and Their Relation to Dog Population Growth

The control and elimination of dog-mediated rabies is reliant not only on vaccination, but also to some extent on humane dog population management (HDPM), in order to achieve herd immunity [25]. In this regard, the survival and proliferation of free-roaming dogs is relevant. Free-roaming dogs most commonly depend on the availability of food, shelter, and freedom of movement, all of which result from human negligence and irresponsibility—particularly as it relates to access to garbage as a source of food [25].

Fast growing dog populations are strongly linked to urbanization and the associated increased solid waste production and mismanagement of waste disposal [26]. The human:dog ratio in urban Asia and urban Africa is estimated to be around 7.5:1 and 21.2:1 [27], but this ratio could be higher in areas surrounding urban dumping sites due to excessive movements of dogs towards the urban dumping site in search of feeding opportunities [28].

The United Nations estimated that the global urban dwelling population would reach up to 68% of the global population by 2050 (compared to the current 55% urban dwelling population) [29]. Countries in Africa and Asia are expected to experience a rapid increase in urbanization rates in the coming years. The increase in human population as well as continuing urbanization is expected to add approximately 2.5 billion individuals to the urban population by 2050, with the majority of the growth occurring in Africa and Asia [29]. Some countries (i.e., India, China, and Nigeria) with the highest projected urban population increase also lead in numbers of dog-mediated human rabies deaths in Asia and Africa, and when combined are likely to add almost 900 million urban dwellers in the coming 30 years [29]. With this increasing trend of urbanization, solid waste production is set to rise as urban dwellers generate two to three fold more municipal waste (in kilogram of waste per capita per day) than rural residents [30].

Generally, rapid immigration to urban areas with a vision of better job opportunities, better schooling, and consequently better life has globally escalated the number of people residing in slums areas and in temporary/makeshift homes. This immigration and urbanization contribute significantly to the accumulation of waste and waste management problems [31]. In this regard, the global annual waste generated in 2016 was 2.01 billion tons—which is estimated to reach 3.4 billion tons per year by 2050. Sub-Saharan and South Asian regions are expected to triple and double their waste production, respectively [32]. This dramatic increase in waste production often leaves governments and local municipalities unable to provide effective waste removal and managements services, in turn leading to waste accumulation in residential areas and open dumping grounds [30].

The major component of municipal solid waste (MSW) is organic biodegradable waste (70%) that serves as a source of food to free-roaming dogs, thereby attracting these dogs in the densely populated urban slums [33]. The hunger stricken dogs compete for food, and associated aggression among these dogs also endangers local inhabitants and significantly increases the threat of rabies transmission to humans [34]. Proper management of waste in the streets is essential as part of a holistic strategy to minimize free-roaming dog populations as an animal welfare consideration and in support of rabies control [35].

In rabies-endemic developing countries, waste disposal systems often consist of collecting the waste from the source and dumping into the nearest open space, as the concepts of recycling and reusing are still in incipient stages [33,36]. Moreover, in metropolitan and sub-metropolitan cities, the number of available landfills has declined, and the local authorities are compelled to dispose of a large amount of MSW in small spaces closer to residential areas [33]. Individuals working at these landfill sites are at high risk of being bitten by foraging stray dogs [37].

## 3. Examples of Rabies Endemic Countries in Asia with High MSW Levels

Rapid urbanization, coupled with an increase in population numbers have dramatically accelerated the rate of MSW generation in most developing countries [36]. The majority of developing countries in Asia is no exception. MSW management in these low and middle-income countries is inefficient and leads to large amounts of waste remaining accessible in open dump sites or on the streets in residential areas [33]. Free-roaming dog densities vary among countries and cities, but very high dog densities (as high as 719 dogs per km^2^) have been reported in some cities in Asian countries [38]. These large numbers of free-roaming dogs are often associated with zoonotic disease transmission to humans [38]. Since the majority of countries in Asia are canine-rabies endemic, large numbers of unvaccinated free-roaming dogs could hamper rabies control efforts.

### 3.1. Nepal

Kathmandu Valley (Nepal) is inhabited by 29 million humans with an annual population growth rate of 1.85% [39,40]. It is estimated that more than 22,000 street dogs are present in the Kathmandu Valley [5]. Around 100–150 cases of dog bites are reported in Shukraraj Tropical and Infectious Disease Hospital at Teku, Kathmandu, alone [5]. In 2010, the average MSW generated in Kathmandu was 523.8 metric ton (t) per day, where street litter was about 69.3t per day and organic material comprised the majority of household waste products [41]. The presence of street litter containing large amounts of organic matter (i.e., kitchen waste) attracts dogs and increases the dog population in that area. Reduction of food waste through actions such as composting or restricting access of dogs to human food waste may aid to decrease the number of street dogs from the locality [42].

### 3.2. India

India has the highest number of dog-mediated rabies deaths in the world [43], with an estimated 20,500 human rabies cases reported each year [44]. Although there is no official data, it is estimated that there are more than 30–35 million street dogs in India, with 1.75 million dog bites reported each year. Delhi, the capital of India, alone has around 400,000 street dogs [45]. More than 100 dog bite cases are reported in Delhi every day, but this number excludes all the cases from private hospitals and clinics [46,47]. In Delhi, around 557 thousand tons of MSW is illegally dumped in streets, roads, and open areas [48].

Chandigarh, India, with a human population of 1.5 million people, generated 360 tons of MSW in 2014 [49]. Incidentally, in 2019, the MSW in Chandigarh increased to 470 tons, with 74% dumped in the dumping ground of the waste processing plant in Dadumajra [50]. In the year 2012, the dog population was estimated to be 17,912, with 6900 bite cases reported [51]. By 2018, the stray dog population had increased to 23,000, with more than 10,000 cases of dog bites reported [51].

Due to poor collection and transportation practices waste accumulates in cities [52]. Only 28% of the total 150,000 t waste that is generated per day is processed in India, where only eight out of 35 states have more than 50% waste processing, and approximately 10 states process less than 10% of the generated solid waste [53]. West Bengal and Karnataka, with waste processing of 32% and 5%, respectively, are jointly responsible for 58% of reported rabies deaths in India [46,54]. Likewise, Uttar Pradesh, which ranks third in municipal waste generation with 15,228t per day, witnesses dog bite cases that affect 2,700,000 people each year [54,55].

Together, this abysmal record of waste control, an estimated national dog population density of 970 dogs/km^2^, along with a low vaccination coverage of about 30% [56], underlines the significance of the challenge to better control and eventually eliminate rabies in India.

### 3.3. Bangladesh

Bangladesh is a densely populated rabies endemic country of the South Asian region that is home to about 1.6 million dogs, where 83% live on the streets [57,58]. The waste generation in Bangladesh was 16,382 ton/day in 2004, which increased to 23,668 tons/day in 2014 and is expected to increase by 100% by 2025 [59,60]. The country reported 50,000 to 200,000 animal bites per year in several years, with almost 84% of bites due to stray and community dogs [61,62,63]. It is currently estimated that on average, there are 2100 human rabies deaths in Bangladesh per year [63]. With nearly half of the MSW generated remaining uncollected, and the majority of the edible organic waste dumped in landfills, ample feeding opportunity for large numbers of free-roaming dogs is created, imposing a dog rabies transmission threat [59].

### 3.4. Pakistan

Likewise, in Pakistan, municipal solid waste increased from approximately 10,000 tons per day in 2015 to 16,000 tons per day currently, where 60% is dumped in landfills, with the remaining portion occupying the streets with no further formal removal taking place [64,65,66]. Various hospitals admit 25–30 dog-bite cases per day in Pakistan, with Indus hospital in Karachi reporting a rise in dog bite cases by more than 200% from 2012 to 2017 [67]. Given that Pakistan is a dog rabies endemic country with some of the highest reported number of rabies cases [68], this increase in dog bites along with the availability of MSW as food sources for dogs increases the risk of rabies transmission from dogs to humans.

## 4. Examples of Rabies Endemic Countries in Africa with High MSW Levels

Similar to the situation in Asia, numerous African countries face problems with MSW management and efficient garbage disposal [69]. Africa is second to Asia in the number of rabies cases. At present, no countries in Africa are free of dog rabies, and the number of cases is underreported [70].

### 4.1. Kenya

Kenya has an approximated dog population of five million animals, of which only 20% are reported to be stray dogs. Despite the majority of dogs in Kenya being owned, these animals are allowed to roam freely, and in the period from 2011 to 2015, 6720 dog bite cases were reported, and 858 human rabies cases were reported from 2002 to 2012 [71]. Low rabies vaccination rates in owned dogs (maximum of 29%) were previously reported for Kenya [71].

In the same time period, Kenya generated around 4950 tons of solid waste per day in 2011, which increased to 5600 tons per day in 2015. The amount of MSW that is collected can range from 80% to as little as 20%, with no waste collection in the slums being reported [72,73]. This increase in MSW coupled with the low collection levels in certain areas again provide access to food sources for free-roaming animals that are not vaccinated against rabies and thus provide increased disease transmission opportunities.

### 4.2. Nigeria

Nigeria, with an expected increase of its urban population of 189 million by 2050, reported 61.1% rabies cases among total dog bites from 2005–2014 [74,75]. Several reports claim an increase in dog bite instances in Nigeria, where 63.7% of the bite incidences have been recorded to be by free-roaming dogs [76,77]. Hamlobu and colleagues reported approximately equal numbers of stray and owned dogs, with 36% of owned dogs allowed to interact with the street dogs [78]. The recommended 70% vaccination coverage in dogs has not been attained, and research indicates high levels of poverty among the population, which may lead to dog dependency on food resources at garbage sites, thus providing possible opportunity for increased disease transmission among dogs and from dogs to humans [78]. Rapid population growth in the country has also increased the amount of waste production—the net MSW density increased from 0.65 Kg/capita/day in 2009 to 0.95 Kg/capita/day (equivalent to 42 million tons annual MSW production) in 2018 [79,80]. The MSW, containing up to 52% organic waste, may sustain large free-roaming dog populations, further enabling the spread of rabies in communities [80].

### 4.3. Tanzania

Tanzania is a dog rabies endemic country with an estimated 2,316,000 dogs and more than 1500 annual rabies deaths, with higher bite incidence reported in rural areas as compared to the urban areas [7,81,82]. The dog rabies elimination demonstration project from 2010–2016 had reduced the number of rabies cases by 75%, through implementation of educational campaigns, increased surveillance efforts in both the human and animal health sectors, and increased dog vaccination, respectively [81]. Contrastingly, only 18% of dogs, responsible for 2500 dog bites in the Kilimanjaro region from 2013–2017, were found to be vaccinated [83]. Even though scavenging habits support possible rabies transmission, 50% of waste is not disposed of properly in Tanzania. In 2006, 39,000 tons/year of industrial waste was produced, with the food and beverage industry responsible for 91% of the total waste generated [84], which increased to 10,000 tons/day in 2020 [85]. It is reported that 1,196,900 (57%) and 625,000 (29.7%) of the total 2,101,500 tons of waste is generated from the household and market places, and 80% of the waste is disposed in open dumpsites where free-roaming dogs feed [85].

### 4.4. Cameroon

In Cameroon, the increasing population in cities such as Yaoundé, coupled with extreme poverty, has increased the solid waste from 850 g/capita/day in 1998 to 6.5 Kg/capita/day in 2007 in Yaoundé alone [86]. As in other developing countries, MSW collection is inefficient, with 30% of waste remaining in open garbage dumps surrounding households, thereby providing food sources for free-roaming dogs and leaving children vulnerable to dog bites during disposal of these wastes into open garbage dumps [86]. Open garbage dumps and market places contributed 68.1% and 18.3% of food sources for free-roaming dogs in Cameroon [86]. As a dog rabies endemic African country, Cameroon reported 30–45 human deaths from rabies in the period 1990 to 1995 [87]. During a rabies surveillance pilot project from 2014–2016, 718 dog bite cases were recorded, with more than 65% having the risk of rabies transmission and only 12.6% of the dogs vaccinated [88].

## 5. Rabies Free and Rabies Endemic Countries, What Makes the Difference?

There are 70 dog rabies-free and 122 dog rabies-endemic countries in the world [9]. Only five endemic countries have attained 70% vaccination coverage in dogs, and more than 100 countries have vaccination coverage below 50% [9]. Dog rabies has been eliminated in Western Europe, North America, Latin America, and some Asian countries by synchronous mass dog vaccinations, strict legislative regulations, and animal population management through spay/neuter campaigns and waste management [89]. However, in the remaining dog rabies-endemic countries, implementation of rabies control programs has experienced limited progress.

There is a link between rabies persistence in dog rabies-endemic countries and MSW mismanagement. A large discrepancy exists between the recycling and reuse of MSW in developing and developed countries worldwide. In developing countries, the main focus is on increasing capacity of waste collection and minimizing uncontrolled dumping of MSW. In contrast, developed countries give priority to reducing waste generation and reuse/recycling activities [90]. In developing countries, large volumes of MSW remain accessible as food sources for free-roaming dogs, not only due to large volumes of uncollected waste in these countries, but also due to lack of access control at dump sites, efficient waste disposal, and recycling activities [32]. Dog rabies free countries in Asia, like Japan, Singapore, and Korea, have made immense progress in waste recycling and management through door to door waste collection [91], thermal recovery, fuel recovery, composting of biodegradable waste, easier disassembly, and incineration, whereas in dog rabies endemic countries, it is just the opposite [33], with countries such as Bangladesh and India still in the inchoate stage in terms of reuse and recycling of the generated waste [60].

Likewise, many countries in Europe and America have established power plants to utilize the MSW produced. There are around 512 power plants in Europe with the capacity to incinerate or reuse 90 million tons of solid waste, with the potential of developing 330 more such power plants to reuse a further 50 million tons of waste [92]. Only around 25% of the total waste generated in Europe is dumped in landfills with the majority of waste either composted, recycled or incinerated [92], whereas Asian rabies-endemic countries dump around 85% [93], and African countries dump around 97% of their generated waste. Asian countries recycle only around 15% of generated waste, and in Africa, countries recycle only 4% of the generated waste [94,95]. These landfill sites serve as feeding and breeding grounds for free-roaming dogs. Organic MSW in the streets of Kathmandu and Chandigarh is attracting many dogs, and the high number of dog bites per day in both Kathmandu and Chandigarh indicate the interrelation of waste management and rabies [51,96].

### Waste Generation and Rabies, Where Is the Link?

A large number of owned and community dogs roam freely without any restriction in countries of Africa and South Asia [9]. These dogs are often unvaccinated, and mismanaged biodegradable waste may act as a source of food for those dogs. When dogs feeding on such garbage are threatened by the local children and inhabitants, the risk of bite incidences increases. The urban sprawl has increased the demand for food production, and these organic waste products, when not properly disposed of, provide adequate nutrition at the disposal of free-roaming dog populations, thereby increasing the likelihood of upturns in dog population numbers and associated disease transmission. Unfortunately, studies investigating the correlation between dog bite incidence, dog population growth, and factors that contribute to the increase in population numbers are limited.

Bangladesh reports that only 50% of the total 13,332 tons/day waste generated is collected, and the rest is disposed of in open garbage dumps, where the estimated 1.5 million dogs living in the country can utilize the MSW as an easy and nutritious source of food, thereby sustaining dog populations and even allowing increase in numbers [97,98]. A study in ten Indian metro cities showed a strong correlation between the size of the city’s population, municipal solid waste produced, and dog bites in the particular year; moreover, significant statistical correlation was observed between yearly dog bites and per capita waste generation [37]. MSW is collected by poor and marginalized people to sustain their livelihood, with the majority being children who are unaware of rabies, which provides further support that slum people are at high risk of dog bites and thus rabies transmission [8]. Other similar studies have also highlighted the issue of waste disposal being neglected by municipalities and leading to increased free-roaming dog populations [56,99]. In many regions of India, the poor state of municipal dustbins and trashcans discourage proper waste disposal, with local residents throwing waste arbitrarily in the streets. Free-roaming dogs may rely heavily on these leftovers, but sometimes due to insufficient feeding opportunities, these dogs turn ferocious and present a significant threat to the locals and in particular children [100].

Free-roaming dogs are also kept as pets by slum-dwellers in a symbiotic relationship for food and protection, which provides shelter for free-roaming dogs to raise and nurture their litter. Aggressive behavior may result when dogs feel threatened by an intruder in their territory, biting to protect their pups, to defend their territories, during interferences in courtship, or to defend themselves from the provokers [101].

To effectively control canine rabies, several interventions like synchronous mass dog vaccinations, dog population management, and strict legislation along with community-level awareness are necessary [25]. With the current global level of vaccine production and rapidly increasing dog populations, attaining 70% vaccination coverage to eliminate dog-mediated human rabies by 2030 seems challenging [9]. More than 30 million additional rabies vaccine doses per year are required, and within 13 years, the dog rabies vaccine shortfall will be 7.5 billion doses of vaccine if production level remains similar to the level of 2015 [9].

It must be noted that the mismanagement of MSW is not the only contributing factor for lack of rabies control in dog rabies endemic countries, as rabies-free countries also deal with a large pile of waste in landfills and free-roaming dogs hovering around [102]. Due to access to landfills being controlled, increased reuse/recycling activities, and widespread vaccination of dogs, rabies remains controlled in these dog populations [102].

However, the evidence suggests that MSW does play a significant role, and, for example, the direct increase in rabies transmission and risk for inhabitants of slum areas residing close to landfill sites has been documented [103]. Improved waste management will also reduce the risk of other zoonotic diseases such as human cystic echinococcosis disease [16,104]. Waste management, as part of HDPM, should therefore be part of dog rabies control programs, and this factor is, in our opinion, often neglected.

## 6. The Way Forward

Rabies remains a disease of great public health concern despite the existence of effective tools to control the disease. In general, free-roaming dog populations are indeed the drivers of rabies transmission cycles in the majority of dog rabies-endemic low and middle income countries. In dog rabies-endemic countries, large unmanaged dog populations can be daunting due to often limited resources available for dog vaccination [25]. It is beneficial that rabies control efforts include humane dog population management (HDPM) activities such as spay and neuter programs and community engagement to improve responsible pet ownership. The accompanied reduction in population turnover may allow vaccination coverage to be maintained, as well as improving the welfare and longevity of dogs in these populations [25]. However, present rabies control programs often do not fully appreciate and therefore neglect the role of good waste management in the control of dog populations. Such efforts of community engagement can include waste management strategies to reduce the human–dog conflict. From a welfare perspective, waste management with regard to dogs that are truly dependent on these wastes for survival must be approached carefully. Simply restricting access to the waste immediately, thereby reducing the food sources, without providing alternative food sources (i.e., community feeding stations in areas where dogs are more tolerated) can fuel human–animal conflict due to heightened aggression of hungry dogs searching for food [105]. This in turn will also increase the opportunities for rabies transmission if bite incidences increase. These scenarios highlight the complexities involved in humane dog population management. Nevertheless, humane dog population management (and MSW management as part thereof) should be recognized as a key long-term issue to be addressed in support of large scale vaccination aimed at effectively breaking rabies transmission in at-risk rabies endemic dog populations [25,105].

## Data Availability

No new data were created or analyzed in this study. Data sharing is not applicable to this article.

## References

[1] Rupprecht C.E., Salahuddin N. (2019). Current status of human rabies prevention: Remaining barriers to global biologics accessibility and disease elimination. Expert Rev. Vaccines.

[2] Brunker K., Mollentze N. (2018). Rabies Virus. Trends Microbiol..

[3] Singh R., Singh K.P., Cherian S., Saminathan M., Kapoor S., Reddy G.M., Panda S., Dhama K. (2017). Rabies—Epidemiology, pathogenesis, public health concerns and advances in diagnosis and control: A comprehensive review. Vet. Q..

[4] Hampson K., Coudeville L., Lembo T., Sambo M., Kieffer A., Attlan M., Barrat J., Blanton J.D., Briggs D.J., Cleaveland S. (2015). Estimating the Global Burden of Endemic Canine Rabies. PLoS Negl. Trop. Dis..

[5] Pantha S., Subedi D., Poudel U., Subedi S., Kaphle K., Dhakal S. (2020). Review of rabies in Nepal. One Health.

[6] Kaphle K., Adhikari N., Tariq M. (2020). Fight against rabies in Nepal: Immediate need for government intervention. One Health.

[7] Sambo M., Cleaveland S., Ferguson H.M., Lembo T., Simon C., Urassa H., Hampson K. (2013). The Burden of Rabies in Tanzania and Its Impact on Local Communities. PLoS Negl. Trop. Dis..

[8] Herbert M., Basha R., Thangaraj S. (2012). Community perception regarding rabies prevention and stray dog control in urban slums in India. J. Infect. Public Health.

[9] Wallace R.M., Undurraga E.A., Blanton J.D., Cleaton J., Franka R. (2017). Elimination of Dog-Mediated Human Rabies Deaths by 2030: Needs Assessment and Alternatives for Progress Based on Dog Vaccination. Front. Vet. Sci..

[10] Conan A., Akerele O., Simpson G., Reininghaus B., Van Rooyen J., Knobel D. (2015). Population Dynamics of Owned, Free-Roaming Dogs: Implications for Rabies Control. PLoS Negl. Trop. Dis..

[11] Massei G., Fooks A.R., Horton D.L., Callaby R., Sharma K., Dhakal I.P., Dahal U. (2017). Free-Roaming Dogs in Nepal: Demographics, Health and Public Knowledge, Attitudes and Practices. Zoonoses Public Health.

[12] Zhang J., Jin Z., Sun G.-Q., Zhou T., Ruan S. (2011). Analysis of Rabies in China: Transmission Dynamics and Control. PLoS ONE.

[13] Zinsstag J., Lechenne M., Laager M., Mindekem R., Naïssengar S., Oussiguéré A., Bidjeh K., Rives G., Tessier J., Madjaninan S. (2017). Vaccination of dogs in an African city interrupts rabies transmission and reduces human exposure. Sci. Transl. Med..

[14] Kartal T., Rowan A.N. (2018). Stray Dog Population Management. Field Man. Small Anim. Med..

[15] Minghui R., Stone M., Semedo M.H., Nel L.H. (2018). New global strategic plan to eliminate dog-mediated rabies by 2030. Lancet.

[16] Otranto D., Dantas-Torres F., Mihalca A.D., Traub R.J., Lappin M., Baneth G. (2017). Zoonotic Parasites of Sheltered and Stray Dogs in the Era of the Global Economic and Political Crisis. Trends Parasitol..

[17] Day M.J., Horzinek M.C., Schultz R.D., Squires R.A. (2016). WSAVA Guidelines for the vaccination of dogs and cats. J. Small Anim. Pract..

[18] Harischandra P.A., Gunesekera A., Janakan N., Gongal G., Abela-Ridder B. (2016). Sri Lanka takes action towards a target of zero rabies death by 2020. WHO South-East Asia J. Public Health.

[19] Milby L. (2015). Volunteering with the WVS in India. Vet. Nurs. J..

[20] Acharya K.P., Acharya N., Phuyal S., Upadhyaya M., Lasee S. (2020). One-health approach: A best possible way to control rabies. One Health.

[21] FEDIAF (2018). European Facts & Figures. http://www.fediaf.org/images/FEDIAF_Facts__and_Figures_2018_ONLINE_final.pdf.

[22] Bedford E. (2019). Number of Dogs in the United States from 2000 to 2017. Statista. American Pet Products Association. https://www.statista.com/statistics/198100/dogs-in-the-united-states-since-2000/.

[23] Bhalla S.J., Kemmers R., Vasques A., Vanak A.T. (2020). ‘Stray Appetites’: A Socio-Ecological Analysis of Free-Ranging Dogs Living Alongside Human Communities in Bangalore, India. bioRxiv.

[24] Jaganmohan M. (2018). Population of pet dogs India 2014–2023. Statista. https://www.statista.com/statistics/1061130/india-population-of-pet-dogs/.

[25] Taylor L.H., Wallace R.M., Balaram D., Lindenmayer J.M., Eckery D.C., Mutonono-Watkiss B., Parravani E., Nel L.H. (2017). The Role of Dog Population Management in Rabies Elimination—A Review of Current Approaches and Future Opportunities. Front. Vet. Sci..

[26] Krystosik A., Njoroge G., Odhiambo L., Forsyth J.E., Mutuku F., LaBeaud A.D. (2020). Solid Wastes Provide Breeding Sites, Burrows, and Food for Biological Disease Vectors, and Urban Zoonotic Reservoirs: A Call to Action for Solutions-Based Research. Front. Public Health.

[27] Knobel D.L., Cleaveland S., Coleman P.G., Fèvre E.M., Meltzer M.I., Miranda M.E.G., Shaw A., Zinsstag J., Meslin F.-X. (2005). Re-evaluating the burden of rabies in Africa and Asia. Bull. World Health Organ..

[28] Tiwari H.K., Robertson I.D., O’Dea M., Vanak A.T. (2019). Demographic characteristics of free-roaming dogs (FRD) in rural and urban India following a photographic sight-resight survey. Sci. Rep..

[29] (2018). World Urbanization Prospects 2018: Highlights.

[30] Tantanee S., Hantrakul S. (2019). Municipal Waste Management Challenge of Urbanization: Lesson Learned from Phitsanulok, Thailand. Geogr. Technol..

[31] Mian M., Zeng X., Nasry A.A.N.B., Al-Hamadani S.M.Z.F. (2017). Municipal solid waste management in China: A comparative analysis. J. Mater. Cycles Waste Manag..

[32] Kaza S., Yao L., Bhada-Tata P., Van Woerden F. (2018). What a Waste 2.0: A Global Snapshot of Solid Waste Management to 2050.

[33] Visvanathan C., Adhikari R., Ananth A.P. 3R Practices for Municipal Solid Waste Management in Asia. Kalmar ECO-TECH ’07 Second Balt Symp Environ Chem. http://faculty.ait.ac.th/visu/public/uploads/ProfVisu%27sCV/Conferance/3/Visvanathan_Kalmar07.

[34] Brookes V.J., Ward M.P., Rock M., Degeling C. (2020). One Health promotion and the politics of dog management in remote, northern Australian communities. Sci. Rep..

[35] Kato M., Yamamoto H., Inukai Y., Kira S. (2003). Survey of the stray dog population and the health education program on the prevention of dog bites and dog-acquired infections: A comparative study in Nepal and Okayama Prefecture, Japan. Acta Med. Okayama.

[36] Guerrero L.A., Maas G., Hogland W. (2013). Solid waste management challenges for cities in developing countries. Waste Manag..

[37] Chandran R., Azeez P.A. (2016). Stray dog menace: Implications and management. Econ. Polit. Wkly..

[38] Smith L., Hartmann S., Munteanu A.M., Villa P.D., Quinnell R., Collins L.M., Villa D. (2019). The Effectiveness of Dog Population Management: A Systematic Review. Animals.

[39] United Nations Population Fund (2017). Population Situation Analysis of Nepal (With Respect to Sustainable Development). Unfpa Nepal. https://nepal.unfpa.org/sites/default/files/pub-pdf/NepalPopulationSituationAnalysis.pdf.

[40] (2020). Worldometer Nepal population. https://www.worldometers.info/world-population/nepal-population/.

[41] Dangi M.B., Pretz C.R., Urynowicz M.A., Gerow K.G., Reddy J. (2011). Municipal solid waste generation in Kathmandu, Nepal. J. Environ. Manag..

[42] Li I. (2019). Management Plan for Stray Dog (Canis lupus familiaris) Populations in Kathmandu, Nepal. Bachelor’s Thesis.

[43] World Health Organization (2018). WHO Expert Consultation on Rabies: Third Report.

[44] Sudarshan M., Madhusudana S., Mahendra B., Rao N., Narayana D.A., Rahman S.A., Meslin F.-X., Lobo D., Ravikumar K. (2007). Gangaboraiah Assessing the burden of human rabies in India: Results of a national multi-center epidemiological survey. Int. J. Infect. Dis..

[45] Tiwari H.K. (2019). Free Roaming Dog Population, Community Perception and Control of Dog Related Rabies: The Indian Story.

[46] Sudarshan M.K., Narayana D.H.A. (2019). Appraisal of surveillance of human rabies and animal bites in seven states of India. Indian J. Public Health.

[47] Singh P. Delhi Sees 120 Cases of Dog Bite Daily in Three Years, But No Stray Count After 2009 | Delhi News—Times of India. The Times of India. https://timesofindia.indiatimes.com/city/delhi/city-sees-120-cases-of-dog-bite-daily-in-3-years-but-no-stray-count-after-2009/articleshow/77439315.cms.

[48] Nagpure A.S. (2019). Assessment of quantity and composition of illegal dumped municipal solid waste (MSW) in Delhi. Resour. Conserv. Recycl..

[49] Rana R., Ganguly R., Gupta A.K. (2015). An assessment of solid waste management system in Chandigarh City, India. Electron J. Geotech. Eng..

[50] Sharma K.D., Jain S. (2019). Overview of Municipal Solid Waste Generation, Composition, and Management in India. J. Environ. Eng..

[51] Gupta N., Gupta R.K. (2019). Animal Welfare and Human Health: Rising Conflicts over Stray Dogs in Chandigarh. South Asia Res..

[52] Kaushal R.K., Chabukdhara M., Varghese G.K. (2012). Municipal Solid Waste Management in India-Current State and Future Challenges: A Review. Int. J. Eng. Sci. Technol..

[53] Malav L.C., Yadav K.K., Gupta N., Kumar S., Sharma G.K., Krishnan S., Rezania S., Kamyab H., Pham Q.B., Yadav S. (2020). A review on municipal solid waste as a renewable source for waste-to-energy project in India: Current practices, challenges, and future opportunities. J. Clean. Prod..

[54] Jadhav R. 75% of Municipal Garbage in India Dumped Without Processing. The Times of India. https://timesofindia.indiatimes.com/india/75-of-municipal-garbage-in-india-dumped-without-processing/articleshow/65190477.cms.

[55] Priyadarshi H., Priya S., Jain A., Khursheed S., Ameen H., Jamiolkowski M., Manassero M., Shehata H. (2020). A Literature Review on Solid Waste Management: Characteristics, Techniques, Environmental Impacts and Health Effects in Aligarh City, Uttar Pradesh, India. Recent Thoughts in Geoenvironmental Engineering, Proceedings of the 3rd GeoMEast 2019 International Congress and Exhibition, Cairo, Egypt, 10–14 November 2019.

[56] Belsare A.V., Gompper M.E. (2013). Assessing demographic and epidemiologic parameters of rural dog populations in India during mass vaccination campaigns. Prev. Vet. Med..

[57] Raju F.R. Is Bangladesh Finally Moving from Culling to Vaccinating Dogs? Dhaka Tribune. https://www.dhakatribune.com/bangladesh/nation/2019/05/09/is-bangladesh-finally-moving-from-culling-to-vaccinating-dogs.

[58] Rahaman K.S. (2017). Free Roaming Dogs: A Threat to Public Health. Int. J. Epidemiol. Res..

[59] Chowdhury T.A., Afza S.R. (2006). Waste Management in Dhaka City-a Theoretical Marketing Model. BRAC Univ. J..

[60] Alam O., Qiao X. (2020). An in-depth review on municipal solid waste management, treatment and disposal in Bangladesh. Sustain. Cities Soc..

[61] Tenzin T., Ahmed R., Debnath N.C., Ahmed G., Yamage M. (2015). Free-Roaming Dog Population Estimation and Status of the Dog Population Management and Rabies Control Program in Dhaka City, Bangladesh. PLoS Negl. Trop. Dis..

[62] Ghosh S., Chowdhury S., Haider N., Bhowmik R.K., Rana S., Marma A.S.P., Hossain M.B., Debnath N.C., Ahmed B. (2016). Awareness of rabies and response to dog bites in a Bangladesh community. Vet. Med. Sci..

[63] Masud M.A., Islam M.H., Adnan M.I., Oh C. (2020). Dog Rabies in Dhaka, Bangladesh, and Implications for Control. Processes.

[64] Abbasi H.N., Xiwu L., Zhao G. (2015). An Overview of Karachi Solid Waste Disposal Sites and Environs. J. Sci. Res. Rep..

[65] Sabir W., Waheed S.N., Afzal A., Umer S.M., Rehman S. (2016). A Study of Solid Waste Management in Karachi City. J. Educ. Soc. Sci..

[66] (2020). A Study for Better Solid Waste Management in Karachi. c40 Cities. https://www.c40.org/case_studies/karachi-swm-study.

[67] Ilyas F. (2018). Anti-Rabies Project Launched in Karachi. Dawn. https://www.dawn.com/news/1382541.

[68] Khan A., Ayaz R., Mehtab A., Naz K., Haider W., Gondal M.A., Umer M., Afzal M.S., Shah N.A., Yayi G. (2019). Knowledge, Attitude & Practices (KAPs) Regarding Rabies Endemicity Among the Community Members, Pakistan. Acta Trop..

[69] Loukil F., Rouached L. (2020). Waste collection criticality index in African cities. Waste Manag..

[70] Nel L. (2013). Discrepancies in Data Reporting for Rabies, Africa. Emerg. Infect. Dis..

[71] Ngugi J.N., Maza A.K., Omolo O.J., Obonyo M. (2018). Epidemiology and surveillance of human animal-bite injuries and rabies post-exposure prophylaxis, in selected counties in Kenya, 2011–2016. BMC Public Health.

[72] Bello I., bin Ismail M.N. (2016). Solid Waste Management in Africa: A Review. Int. J. Waste Resour..

[73] Waweru S., Kanda E.K. Municipal Solid Waste Management in Kenya: A Comparison of Middle Income and Slum Areas. Proceedings of the International Conference on Disaster Risk Reduction & Conflict Resolution for Sustainable Development.

[74] Ishaya T., Ibironke O., Stella I., Olatunde A., Gyang M., Israel B., Saidu J., Gambo R., Peterside K., Christianah A. (2016). Dog Bites and Rabies: A Decade Perspective in Nigeria (2005–2014). Worlds Vet. J..

[75] Avis W. (2019). Urban Expansion in Nigeria.

[76] Adeleke S.I. (2010). Impact of dog bite in kano city a retrospective study. Niger. J. Clin. Pract..

[77] Hambolu S.E., Dzikwi A.A., Kwaga J.K.P., Kazeem H.M., Umoh J.U., Hambolu D.A. (2013). Rabies and Dog Bites Cases in Lagos State Nigeria: A Prevalence and Retrospective Studies (2006–2011). Glob. J. Health Sci..

[78] Hambolu S.E., Dzikwi A.A., Kwaga J.K., Kazeem H.M., Umoh J.U., Hambolu D.A. (2013). Dog Ecology and Population Studies in Lagos State, Nigeria. Glob. J. Health Sci..

[79] Ogwueleka T. (2009). Municipal Solid Waste Characteristics and Management in Nigeria. J. Environ. Health Sci. Eng..

[80] Ike C.C., Ezeibe C.C., Anijiofor S.C., Daud N.N.N. (2018). Solid Waste Management in Nigeria: Problems, Prospects, and Policies. J. Solid. Waste Technol. Manag..

[81] Mpolya E.A., Lembo T., Lushasi K., Mancy R., Mbunda E.M., Makungu S., Maziku M., Sikana L., Jaswant G., Townsend S. (2017). Toward Elimination of Dog-Mediated Human Rabies: Experiences from Implementing a Large-scale Demonstration Project in Southern Tanzania. Front. Vet. Sci..

[82] Sambo M., Hampson K., Changalucha J., Cleaveland S., Lembo T., Lushasi K.S., Mbunda E., Mtema Z., Sikana L., Johnson P.C.D. (2018). Estimating the Size of Dog Populations in Tanzania to Inform Rabies Control. Vet. Sci..

[83] Mtui-Malamsha N., Sallu R., Mahiti G.R., Mohamed H., OleNeselle M., Rubegwa B., Swai E.S., Makungu S., Otieno E.G., Lupindu A.M. (2019). Ecological and Epidemiological Findings Associated with Zoonotic Rabies Outbreaks and Control in Moshi, Tanzania, 2017–2018. Int. J. Environ. Res. Public Health.

[84] Mbuligwe S.E., Kaseva M.E. (2006). Assessment of industrial solid waste management and resource recovery practices in Tanzania. Resour. Conserv. Recycl..

[85] Nyampundu K., Mwegoha W.J.S., Millanzi W.C. (2020). Sustainable solid waste management Measures in Tanzania: An exploratory descriptive case study among vendors at Majengo market in Dodoma City. BMC Public Health.

[86] Raymond T.N., Roland M.E., Francoise K.F.M.M., Francis Z., Livo E.F., Clovis S.T.H. (2015). Do open garbage dumps play a role in canine rabies transmission in Biyem-Assi health district in Cameroon?. Infect. Ecol. Epidemiol..

[87] Awah N.J., Tchoumboue J., Tong J. (2002). Canine and Human Rabies in Cameroon. Trop. Vet..

[88] Sofeu C.L., Broban A., Njimah A.N., Momo J.B., Sadeuh-Mba S.A., Druelles S., L’Azou M., Tejiokem M.C. (2018). Improving systematic rabies surveillance in Cameroon: A pilot initiative and results for 2014–2016. PLoS Negl. Trop. Dis..

[89] Vigilato M.A.N., Clavijo A., Knobl T., Silva H.M.T., Cosivi O., Schneider M.C., Leanes L.F., Belotto A.J., Espinal M.A. (2013). Progress towards eliminating canine rabies: Policies and perspectives from Latin America and the Caribbean. Philos. Trans. R. Soc. Lond. B Biol. Sci..

[90] Cervantes D.E.T., Martínez A.L., Hernández M.C., De Cortázar A.L.G. (2018). Using indicators as a tool to evaluate municipal solid waste management: A critical review. Waste Manag..

[91] Zheng P., Zhang K., Zhang S., Wang R., Wang H. (2017). The door-to-door recycling scheme of household solid wastes in urban areas: A case study from Nagoya, Japan. J. Clean. Prod..

[92] Scarlat N., Fahl F., Dallemand J.-F. (2019). Status and Opportunities for Energy Recovery from Municipal Solid Waste in Europe. Waste Biomass Valorization.

[93] Agamuthu P., Fauziah S.H. (2014). Sustainable 3R Practice in the Asia and Pacific Regions: The Challenges and Issues. Spatial Modeling in Forest Resources Management.

[94] Matter A., Dietschi M., Zurbrügg C. (2013). Improving the informal recycling sector through segregation of waste in the household —The case of Dhaka Bangladesh. Habitat Int..

[95] United Nations Environment Programme (2018). Africa Waste Management Outlook.

[96] Duwal B.L. (2020). Condition of Stray Dogs Critical. The Himalayan Times. https://thehimalayantimes.com/lifestyle/condition-of-stray-dogs-critical/.

[97] Zahur M., Otoma S. Informal Waste Recycling Activities: A Case Study of Dhaka City, Bangladesh. Proceedings of the 24th Annual Conference of Japan Society of Material Cycles and Waste Management.

[98] Chowdhury A.H., Mohammad N., Haque M.R.U., Hossain D.T. (2014). Developing 3Rs (Reduce, Reuse and Recycle) Strategy for Waste Management in the Urban Areas of Bangladesh: Socioeconomic and Climate Adoption Mitigation Option. IOSR J. Environ. Sci. Toxicol. Food Technol..

[99] Assa M. (2013). Emerging Solid Waste Market in Lilongwe Urban, Malawi: Application of Dichotomous Choice Contingent Valuation Method. J. Sustain. Dev. Afr..

[100] Bhanganada K., Wilde H., Sakolsataydorn P., Oonsombat P. (1993). Dog-bite injuries at a Bangkok teaching hospital. Acta Trop..

[101] Miller K.A., Dolan E.D., Cussen V.A., Reid P.J. (2019). Are underweight shelter dogs more likely to display food aggression toward humans?. Animals.

[102] Mukherjee C., Denney J., Mbonimpa E.G., Slagley J., Bhowmik R. (2020). A review on municipal solid waste-to-energy trends in the USA. Renew Sustain. Energy Rev..

[103] Kumar N., Singh A., Harriss-White B. (2019). Urban waste and the human-animal interface in Delhi. Econ. Polit. Wkly..

[104] Mandal S., Deb Mandal M. (2012). Human cystic echinococcosis: Epidemiologic, zoonotic, clinical, diagnostic and therapeutic aspects. Asian Pac. J. Trop. Med..

[105] (2019). Humane Dog Population Management Guidance.

